# Practice of Medicine

**Published:** 1893-12

**Authors:** 

**Affiliations:** Professor of Pathology and Lecturer on Mental and Nervous Diseases in the Medical Department of the University of Texas, Galveston


### Current Medical Literature.

### DEPARTMENT OF PRACTICE OF MEDICINE.

EDITED BY ALLEN J. SMITH, M. D., GALVESTON,

Professor of Pathology and Lecturer on Mental and Nervous Diseases in the Medical Department of the University of Texas, Galveston.

---

TUFT'S MEDICAL COLLEGE.—The trustees of Tuft's College have decided to establish a medical department, which has been located in Boston. Males and females are admitted on equal footing.

---

PROF. J. M. CHARCOT, the great neurologist of Paris, died on August 17, 1893, in the sixty-eighth year of his life. He was born in 1825, graduated in medicine in 1853, and became a member of the Institute of France in 1883.

---

DR. WILLIAM B. TOWLES, formerly professor of anatomy and materia medica in the University of Virginia, died on the 15th of September, last, after a very brief illness. His position has been assumed by Dr. Christian, formerly the demonstrator of anatomy in the same institution.

---

TREATMENT OF CARBUNCLES BY CARBOLIC ACID INJECTIONS.—Dr. C. H. Wilkinson, of Galveston (*Med. and Surg. Reporter*, September 16, 1893), reports a case of carbuncle cured by interstitial injection of carbolic acid. The writer urges the value of this method of treatment as second in its curative effects only to the knife, as more easily performed than a cutting operation, and as preferable because of its painlessness. He usually adds ten minims of pure carbolic acid to as many drops each of glycerine and alcohol. One grain of cocaine might be added to this mixture to insure absence from pain, but this is scarcely necessary, as the acid soon acts as a local anæsthetic, the smarting pain lasting but a moment. Twenty minims of this mixture are injected through an ordinary hypodermic syringe, or through a Heaton's hernia needle, into the carbuncle, right and left, out toward the periphery, until all portions of the growth have been reached. Usually one injection suffices, but it may be found neeessary to repeat in forty-eight hours, if the inflammation has not been allayed. Any excess of the fluid should be taken up by a bit of absorbent cotton, or paper, a soft pad of the former being bound on the surface of the carbuncle.

---

FIRST MEDICAL ORGANIZATION IN THE UNITED STATES.—On July 23, 1766, a large body of physicians met at New Brunswick, New Jersey, and formed themselves into a society to be known as the New Jersey Medical Society; adopted instruments of association and constitution, and elected officers. Regular meetings were held twice a year, and the original records of these are in the possession of the present State Society. In 1781 this association petitioned the Provincial Assembly to pass an act regulating the practice of medicine, and on September 26, 1772, the act was passed. An examination in physics and surgery was provided for, and two judges of the supreme court, with the assistance of such proper persons as they should deem fit, conducting the examination and issuing certificates. Any person practising medicine in the colony without such a certificate (except those licensed by the crown or the other colonies) was liable to a fine. A State charter was obtained in 1790, having been originally applied for in 1774. There was an intermission from 1775 to 1781 in the regular meetings, owing to the war with Great Britain. With this exception, the records of the New Jersey State Medical Society from 1766 to the present are unbroken.—*E. B. Godfrey in Times and Register, Nov. 11, 1893.*

---

CREASOTE IN THE TREATMENT OF PHTHISIS PULMONALIS.—Dr. F. M. Warner (*New Yo1k Medical Journal*, November 11, 1893), states that after an extensive employment of creasote in the treatment of phthisis in hospital and private practice for the past four years, he is persuaded of the positive value of the drug as an agent for combating the effects of tuberculosis. His earlier lack of success has decidedly diminished since the size of the doses employed has increased. He begins with one minim thrice daily, and increases by one minim daily until the limit of tolerance is reached, watching the effect of the remedy on the stomach and bowels, and examining the urine daily. The general condition of the patient usually rapidly improves; at first the cough becomes less and less during the day, remaining unchanged at night, and finally improves at night; any tendency to hemorrhage quickly disappears; the sputum becomes thin and frothy, and contains less solid material; the night sweats grow less; and for a time, at least, the weight increases. These effects do not appear with small doses; they depend on the saturation of the patient with the drug. One patient of the writer's has taken as much as 215 minims daily, with no ill effect. The author advises the simultaneous employment of antiseptic inhalations to aid the treatment, such as creasote with terebene, or ether in fifty per cent. solutions, a few drops on the sponge of a Robinson's inhaler used every two or three hours or oftener. Usually, should there appear symptoms of a toxic nature (nausea, vomiting, gastric pain, diarrhœa, dizziness, painful and double vision), they disappear with diminution of the dose.

---

THE DIAGNOSIS OF TYPHOID FEVER.—A recent number of the *London Lancet* contains, in substance, the following: Dr. V. Filipovich, of Odessa, has remarked that, in all cases of typhoid fever that have come under his notice during the last two extensive epidemics in Odessa, there have been a peculiar induration and yellowish or orange tint in the prominent portions of the plantar and palmar surfaces, instead of the reddish color to be observed in healthy subjects, or the bluish tinge which those parts assume in a cyanotic patient. This yellowish appearance is to be accounted for by the enfeebled action of the heart, the incomplete filling of the capillaries, and the dryness of the skin in typhoid fever. This sign is so constant and so well marked, that Dr. Filipovich thinks that it should be looked upon as affording important assistance in the diagnosis of doubtful cases. That it is in no sense peculiar to the two epidemics that ocurred in Odessa, is shown by the observations of Dr. Skibnevski, who states that he invariably observed it during an epidemic in the neighborhood of Moscow.—*Medical Record, Sep. 23, 1893.*

(For at least three years the editor of this department has taken every opportunity to observe this sign in cases of typhoid fever coming under his notice, and thus far has never found it absent after the first week of the illness. It usually persists, moreover, until the period of convalescence. The symptom was first mentioned to the writer by Dr. S. Solis-Cohen, of Philadelphia, between three and four years ago, with whom its observation was supposed to have been original. The symptom is, however, not entirely peculiar to typhoid fever, being occasionally seen in cases of advanced pulmonary consumption, and other affections of a wasting type attended with heightened temperature. It is probable that the explanation given by Dr. Filipovich is in a measure correct as to the cause of the color of these parts of the body, but it can scarcely be doubted but there is also actual pigmentation of the superficial layers of the skin when the brightness of the tint is seen in well marked cases, in whom it persists when the palms are moist as well as when they are dry. The symptoms should be of some service in the diagnosis of typhoid fever from ordinary cases of continued malarial fever, in the latter of which diseases the yellowness of the surface is generally much more widely distributed, and is not of such a canary, or orange hue.)

---

YELLOW FEVER FROM A MEDICO-GEOGRAPHICAL AND PROPHYLACTIC POINT OF VIEW IN THE MEXICAN REPUBLIC.—The contribution of Prof. Eduardo Liceago, of Mexico (*Medical and Surgical Reporter*, November 4, 1893), read before the recent International Congress of Public Health (October, 1893), is of decided interest to Southern American practitioners. Professor Liceago names the following places as points where yellow fever is almost always present, and which may be considered as centers of infection: Vera Cruz, Alvarado, Tlacotalpan, Laguna, Campeche, Merida, the capital of Yucatan, as well as the following districts of Yucatan, Unucma, Progreso, Temax, Tizimin and Valladolid, and perhaps Motul and Mazcanu. Of the 2580 kilometers of gulf coast, it will thus be seen that but a small part, belonging to the canton of Vera Cruz, the district of Frontera, Campeche and the northern coast of the peninsula of Yucatan, can be regarded as the habitat of yellow fever. Moreover, these last named districts are separated but by the Yucatan canal, a narrow strait, from the island of Cuba, where the fever also reigns. The author suggests this proximity to Cuba as a cause for the especial prevalence of the fever in Yucatan.

2. All the coast of Mexico, on the Gulf of Mexico and on the Pacific, is well adapted for the disease when imported.

3. Yellow fever has become epidemic in the following places of the coast of the gulf: Matamoros, Altamira, Tampico, Tuxpan, Papantla, Misantla, Nautla, Alvarado, Coatzacoalcos, Minatitlan and San Juan Bautista de Tabasco.

4. The fever has extended in an epidemic form into the interior, but never into places situated more than 1008 meters above the level of the sea.

5. On the Pacific coast there is not a single yellow fever center, but it has been imported into the following places: On the peninsula of Lower California—La Paz y Todos Santos; on the continent—Guaymas, Altata, San Blas, Manzanillo, Acaponeta, Acapulco, Puerto Angel, Salina Cruz, Tonala, Soconuzco, Tapachula and San Benito; and in the interior, in Hermosillo y Culiacan.

6. Immunity against yellow fever is obtained after having had the disease in any of its forms. It is possible that this immunity may be lost at times, but it is seldom the case, as happens with typhus, small-pox and scarlet fever, which may be had by a person previously affected.

7. The vaccine of Jenner against small-pox, and others which science has discovered, authorize us to look for the one that will prevent the yellow fever. The inoculations against this fever which Dr. Carmona y Valle has practiced with success, should be tried on a large scale, in a uniform manner, in order to find out if they are efficacious or not. If future experience should confirm them, they should be made compulsory in the countries where the fever reigns. If they should prove unworthy, the plan suggested by Sternberg, of inoculation with the serum from persons enjoying immunity, should be tried on a large scale and in a uniform manner.

8. The purification of the drinking waters used by persons who have to expose themselves to contact with the disease should be recommended. The purification of the water on board the ships leaving or calling at infected ports, should be proposed.

9. The sanitation of the places which are yellow fever centers should be done at once, to prevent by sanitary police measures the importation of the fever into places where it can be developed.

